# Combined effect of papuamine and doxorubicin in human breast cancer MCF-7 cells

**DOI:** 10.3892/ol.2014.2218

**Published:** 2014-06-03

**Authors:** SYU-ICHI KANNO, SHIN YOMOGIDA, AYAKO TOMIZAWA, HIROYUKI YAMAZAKI, KAZUYO UKAI, REMY E.P. MANGINDAAN, MICHIO NAMIKOSHI, MASAAKI ISHIKAWA

**Affiliations:** 1 Department of Clinical Pharmacotherapeutics, Tohoku Pharmaceutical University, Sendai 981-8558, Japan; 2 Department of Natural Product Chemistry, Tohoku Pharmaceutical University, Sendai 981-8558, Japan; 3 Faculty of Fisheries and Marine Science, Sam Ratulangi University, Kampus Bahu, Manado 95115, Indonesia

**Keywords:** papuamine, doxorubicin, colony formation, c-Jun N-terminal kinase

## Abstract

Our previous study reported that an extract of an Indonesian marine sponge, *Haliclona* sp., showed potent cytotoxicity and induced apoptosis. The major cytotoxic chemical compound was identified as papuamine, which caused reduction of cell survival through activation of c-Jun N-terminal kinase (JNK) in human breast cancer MCF-7 cells. Doxorubicin (DOX), a *Streptomyces* metabolite, is used in chemotherapy against a wide range of cancers, including breast cancer. The present study examined the combined effect of papuamine and DOX on MCF-7 cells. The effect of these reagents on cell growth was assessed by a colony formation assay. Incubation with either of the reagents alone resulted in concentration-dependent decreases in the colony formation of the MCF-7 cells. Incubation with the reagents together at sub-cytotoxic concentrations resulted in significant decreases in colony formation. The phosphorylation of JNK, the activated form of the protein, was elevated in a concentration-dependent manner upon co-incubation with papuamine and DOX. Fluorescence intensity analysis demonstrated that papuamine caused a small, but non-significant, decrease in cellular accumulation of DOX. These results indicate that the combinatory effect of papuamine and DOX is not associated with changes in the cellular accumulation of DOX, and may instead reflect additive effects on JNK activation. This study indicates that papuamine may represent a novel type of modulator for DOX chemotherapy.

## Introduction

The long-term survival rate among patients with breast cancer across Europe is >70% (1). This rate is, in part, due to the widespread use of post-surgery adjuvant anthracycline-based chemotherapy to treat early-stage breast cancer to reduce the risk of relapse and mortality (2). The major anthracycline chemotherapeutic reagent is doxorubicin (DOX), a *Streptomyces* metabolite. DOX-based chemotherapy is used against a wide range of cancers, including hematological malignancies, soft-tissue sarcomas, lymphomas and various types of carcinomas, as well as breast cancer, despite the clinical limitations of the compound, such as cardiotoxicity and the induction of multidrug resistance (3,4). In an attempt to address these limitations, DOX therapy in the clinic is often supplemented by DOX in combination with other chemotherapeutic reagents. The cytotoxicity of DOX is, in part, effected through the c-Jun N-terminal kinase (JNK); JNK-dependent signaling plays a prominent role in DOX-induced cell cycle withdrawal, differentiation and the control of apoptosis (5,6). The JNK pathway has been demonstrated to be required for apoptosis caused by chemotherapeutic agents (7). Thus, synergistic apoptotic responses may require JNK signals and could consequently be manipulated for the potentiation of cancer therapies. Our previous study showed that an extract of an Indonesian marine sponge, *Haliclona* sp., demonstrated potent cytotoxicity against multiple human solid cancer cell lines (8). Previous studies on flow cytometric analyses and nuclear morphological changes have indicated that one of the active components of the extract induced apoptosis; the major cytotoxic activity was identified as being caused by papuamine (8). Our subsequent study showed that papuamine inhibits MCF-7 cell survival through the activation of JNK (9). The present study examined the potential modulation of doxorubicin cytotoxicity by papuamine in MCF-7, a human breast cancer cell line.

## Materials and methods

### Chemicals and cell cultures

Papuamine was isolated from Indonesian marine sponge *Haliclona* sp. using our previously published methods (8). Papuamine was dissolved in dimethyl sulfoxide and stored as a 20-mM stock solution in light-proof containers at −20°C. Doxorubicin (DOX) and all other reagents, unless otherwise stated, were of the highest grade available and were supplied by either Sigma (St. Louis, MO, USA) or Wako Pure Chemical Industries, Ltd. (Osaka, Japan). Exposure to light was kept to a minimum for all drugs used. Immunoblotting employed rabbit polyclonal antibodies (Cell Signaling Technology, Inc., Danvers, MA, USA) against human proteins as follows: Anti-JNK (to detect total JNK levels), anti-phospho-JNK (to detect levels of phosphorylated JNK) and anti-β-actin (as the loading control). The MCF-7 human breast cancer cell line was supplied by the Cell Resource Center for Biomedical Research, Tohoku University (Sendai, Japan). The cells were maintained in RPMI-1640 medium supplemented with 10% fetal bovine serum, 100 U/ml penicillin G and 100 μg/ml streptomycin at 37°C in a humidified 5% CO_2_-95% air incubator under standard conditions. Viable cell counts were determined using exclusion of staining by 0.2% trypan blue. To maintain exponential growth, the cells were seeded at 5×10^4^ cells/ml in standard tissue culture flasks and passaged every 3–4 days. For other assays, the cells were cultured in 2-ml aliquots in 35-mm dishes.

### Soft agar colony formation assay

The effect of papuamine and DOX on the colony formation of the MCF-7 cells was assessed by soft agar colony formation assay. The assay was performed in 35-mm dishes; each plate received 2 ml 0.8% agar (in culture medium), which then was overlaid with a top layer of 1 ml 0.4% agar. Next, ~2,000 cells were plated over the top layer. The cells were treated with reagents and maintained at 37°C in a humidified 5% CO_2_ atmosphere. At day 14 post-treatment, the cells were stained by exposure (60 min at 37°C) to 100 μl 5-mg/ml 3-(4,5-dimethylthiazol-2-yl)-2,5-diphenyl tetrazolium bromide. Each dish then was assessed for the size and number of colonies (original magnification, ×10).

### Western blot analysis

The cells were washed with phosphate-buffered saline (PBS) and lysed in CelLytic™ M (Sigma) to collect a total cell lysate, according to the manufacturer’s instructions. Protein concentration was measured using a Pierce™ BCA Protein Assay kit (Thermo Fisher Scientific, Inc., Rockford, IL, USA), according to the manufacturer’s instructions. Following electrophoresis of protein samples (30 μg/lane) on 10% SDS-polyacrylamide gel, the protein was transferred to a polyvinylidene difluoride membrane. The protein was blocked with Blocking One^®^ (Nacalai Tesque, Inc., Kyoto, Japan) for 1 h and incubated with a first antibody overnight at 4°C. The membrane was then washed with wash buffer (PBS containing 0.05% Tween-20) and incubated with anti-rabbit horseradish peroxidase-linked secondary antibody (Cell Signaling Technology, Inc.) for 1 h. Subsequent to another wash with wash buffer, the protein levels were analyzed by enhanced chemiluminescence with Pierce Western Blotting substrate (Thermo Fisher Scientific, Inc.).

### DOX cellular accumulation assay

The cellular accumulation of DOX was ascertained using a Tecan Infinite^®^ M1000 microplate reader (Tecan Group Ltd., Männedorf, Switzerland) at excitation and emission wavelengths of 485 and 590 nm, respectively. Briefly, the cells were seeded at a 5×10^3^/well in Nunc™ MicroWell™ 96-well optical-bottom plates (Thermo Fisher Scientific, Inc.) and incubated overnight under standard culture conditions. Media was replaced with culture medium supplemented with various concentrations of papuamine (1, 2 or 5 μM) and doxorubicin (1, 3, 10 and 30 μM) and cultures were incubated for 4 h. The medium was removed and washed twice with PBS, and the residual fluorescence intensity was measured as aforementioned.

### Statistical analysis

Statistical analysis was performed by one- or two-way analysis of variance followed by Williams’ type multiple comparison or Bonferroni tests among multiple groups. Data are expressed as the mean ± standard deviation. P<0.05 was considered to indicate a statistically significant difference.

## Results

### Inhibition of colony formation by papuamine and DOX

**Figure 1:**
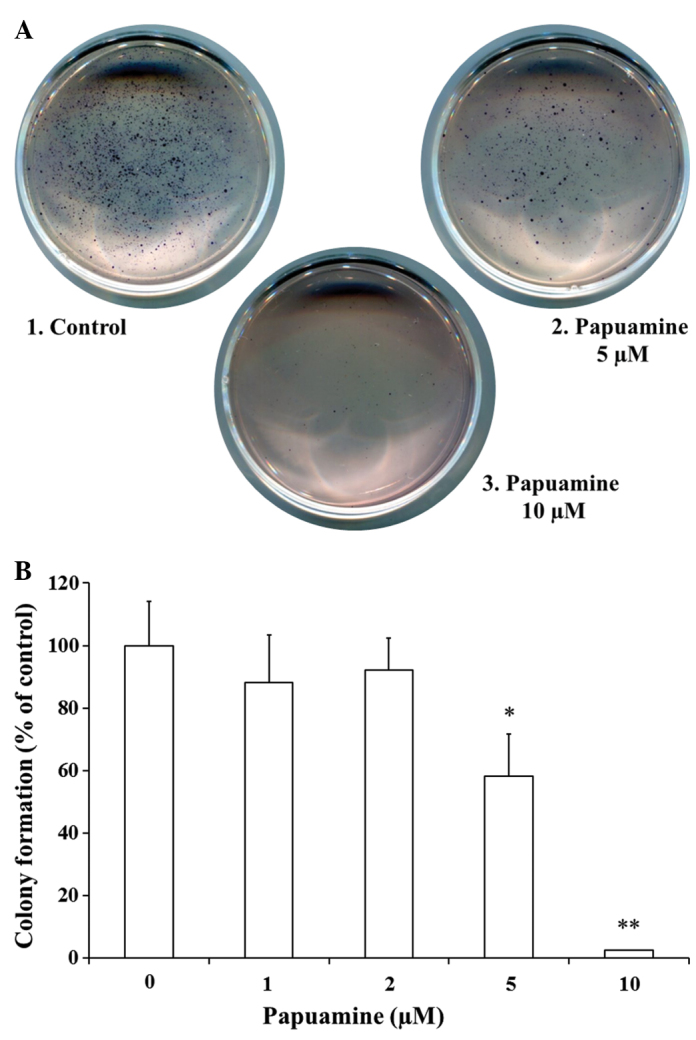
Effect of papuamine on colony formation by MCF-7 cells. To determine the effects on cell growth, colony formation assays were performed in the presence or absence of 1–10 μM papuamine alone. (A) Representative colony formation assay results of incubation with papuamine for 14 days. (B) Colony number was counted following incubation with papuamine for 14 days. Results are the mean ± standard deviation of three individual studies. ^*^P<0.05 or ^**^P<0.01 vs. control group under indicated culture conditions.

**Figure 2:**
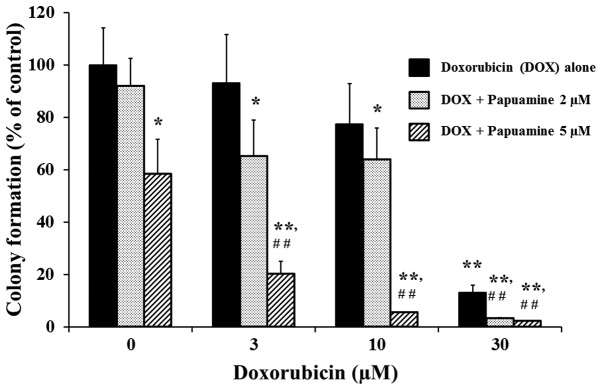
Effect of papuamine and doxorubicin (DOX) on colony formation ability in MCF-7 cells. Colony number was counted following incubation with papuamine and/or DOX for 14 days. Results are the mean ± standard deviation of three individual studies. ^*^P<0.05 or ^**^P<0.01 vs. control group under indicated culture conditions. ^##^P<0.01 vs. incubation with DOX alone under indicated culture conditions.

First, the present study examined whether incubation with papuamine and DOX, alone and in combination, affected the ability of MCF-7 cells to form colonies in soft agar. Following treatment with reagents, the cells were placed into medium with soft agar, and colonies were counted subsequent to 2 weeks. As shown in Fig. 1A and B, incubation with papuamine alone at 5 or 10 μM provided significant concentration-dependent inhibition of colony formation (58.3±13.4%, P<0.05 at 5 μM; 2.1±0.2%, P<0.01 at 10 μM) compared with the control. Papuamine exhibited little effect at lower concentrations of ≤2 μM. As shown in Fig. 2, incubation with DOX alone at 3, 10, or 30 μM provided significant (P<0.01) concentration-dependent inhibition of colony formation (93.2±18.4, 77.4±15.4 and 13.2±3.2%, respectively) compared with the control. These effects were significantly enhanced by co-incubation with papuamine at 2 or 5 μM (P<0.01 compared to single incubation with DOX).

### Detection of JNK activation by papuamine and DOX

**Figure 3:**
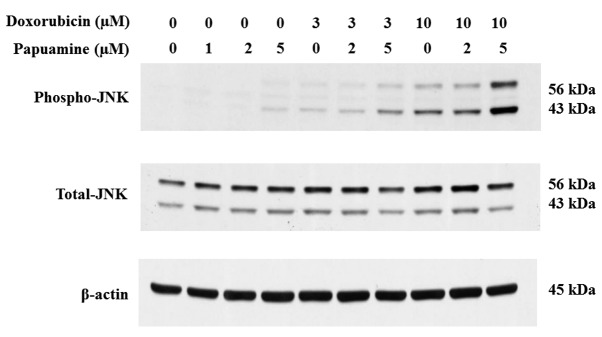
Effects of papuamine and doxorubicin (DOX) on the induction of c-Jun N-terminal kinase (JNK) activation, as assayed by western blotting. The cells were incubated with these reagents for 24 h and samples were prepared as described in the Materials and methods section. The level of the indicated proteins was analyzed by western blot analysis using the level of β-actin as a loading control. The experiments shown are representative of a minimum of three independent experiments.

Next, the study examined the possible involvement of the JNK pathway in the inhibition of cell growth in MCF-7 cells. JNK activation was determined by western blot analysis following a 24-h treatment of the cells with reagents (Fig. 3). Phosphorylated JNK (phospho-JNK), an activated form of the protein, was detected at low levels following incubation with either papuamine (5 μM) or DOX (3 μM) alone. Phospho-JNK levels exhibited concentration-dependent increases upon co-incubation with papuamine and DOX (Fig. 3, upper panel). In control blots, the levels of total JNK and β-actin were not changed under these same conditions (Fig. 3, lower and middle panels).

### Effect of papuamine on DOX cellular accumulation

**Figure 4:**
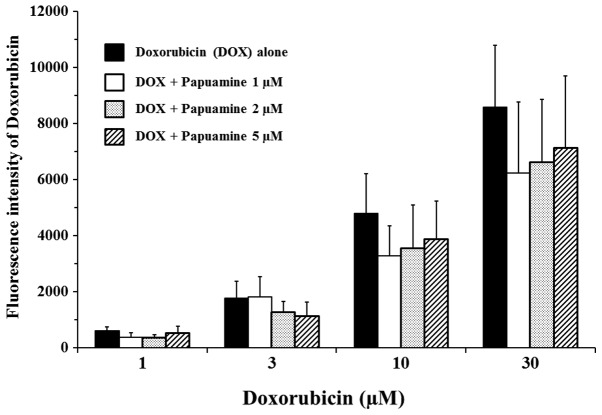
Effect of papuamine on the cellular accumulation of doxorubicin (DOX). The cellular accumulation of DOX incubation for 4 h in the presence or absence of 1–5 μM papuamine was measured by fluorometric analysis (Ex=485 nm and Em=590 nm) as described in the Materials and methods section. Results are the mean ± standard deviation of three individual studies.

The effect of papuamine may be exerted by changes in the cellular uptake or efflux of DOX in MCF-7 cells. Therefore, the effect of papuamine on DOX cellular accumulation was examined using a fluorescence assay (Fig. 4). DOX autofluoresces with λex=485 nm and λem=590 nm. Incubation with DOX alone (at 1 to 30 μM) for 4 h showed concentration-dependent increases in fluorescence intensity. Co-incubation with papuamine at 1, 2 or 5 μM resulted in small (≤25%) decreases in DOX fluorescence. Notably, however, these decreases were not statistically significant or concentration-dependent for papuamine.

## Discussion

To evaluate the effects of papuamine and DOX on cell growth, colony formation assays were performed in soft agar in the present study. Soft agar colony formation assays are commonly used as a monitoring system for anchorage-independent (three-dimensional) growth, measuring proliferation by the counting of colonies subsequent to 3–4 weeks in a semi-solid culture medium. This traditional method has been widely published (10–12). As the technique delivers results that are comparable to those obtained when injecting tumorigenic cells into nude mice, the soft agar colony formation assay is regarded as the gold standard for testing the tumorigenicity of cells *in vitro* (13). Notably, in the present study, incubation with 10 μM papuamine alone provided significant inhibition of colony formation by the MCF-7 cells, while incubation with the same concentration (10 μM) of DOX did not yield significant inhibition, indicating that papuamine was more potent than DOX. Co-incubation with papuamine and DOX enhanced the inhibition of colony formation. Thus, these results indicate potential efficacy for the use of a combined papuamine and DOX chemotherapy regime in the treatment of breast cancer. To confirm the possible mechanisms of the combinatory effects of papuamine and DOX in MCF-7 cells, the activation of JNK and the cellular accumulation of DOX was examined in the treated cells. JNK has previously been indicated to be a key player in the DOX-induced apoptotic cascade (14), and our previous study showed that papuamine-induced cytotoxicity involved JNK activation (9). In the present study, it was demonstrated that these reagents concurrently increased JNK activation, consistent with their additive action in inhibiting colony formation. Previous studies have shown that DOX is a substrate for P-glycoprotein (P-gp), meaning that the inhibition of P-gp could increase the cellular accumulation of DOX and enhance its chemotherapeutic effect (15–17). However, the present study demonstrated that papuamine did not significantly alter DOX cellular accumulation in MCF-7 cells. These results indicate that papuamine does not enhance DOX efficacy by increasing DOX accumulation (as may apply for changes in P-gp), suggesting that papuamine represents a novel type of modulator for DOX chemotherapy.

In conclusion, papuamine and DOX exhibit synergy when used as a combination treatment in MCF-7 cells. This combination effect is not mediated by changes in the cellular accumulation of DOX, but appears to reflect a shared activation of JNK phosphorylation. The present study may therefore aid in the development of therapeutic strategies for breast cancer treatments.
